# Realigning Thunder and Lightning: Temporal Adaptation to Spatiotemporally Distant Events

**DOI:** 10.1371/journal.pone.0084278

**Published:** 2013-12-31

**Authors:** Jordi Navarra, Irune Fernández-Prieto, Joel Garcia-Morera

**Affiliations:** 1 Parc Sanitari de Sant Joan de Déu & CIBERSAM, Fundació Sant Joan de Déu, Esplugues de Llobregat, Barcelona, Catalonia, Spain; 2 Institut de Recerca en Cervell, Cognició i Conducta (IR3C), Universitat de Barcelona, Barcelona, Catalonia, Spain; Bielefeld University, Germany

## Abstract

The brain is able to realign asynchronous signals that approximately coincide in both space and time. Given that many experience-based links between visual and auditory stimuli are established in the absence of spatiotemporal proximity, we investigated whether or not temporal realignment arises in these conditions. Participants received a 3-min exposure to visual and auditory stimuli that were separated by 706 ms and appeared either from the same (Experiment 1) or from different spatial positions (Experiment 2). A simultaneity judgment task (SJ) was administered right afterwards. Temporal realignment between vision and audition was observed, in both Experiment 1 and 2, when comparing the participants’ SJs after this exposure phase with those obtained after a baseline exposure to audiovisual synchrony. However, this effect was present only when the visual stimuli preceded the auditory stimuli during the exposure to asynchrony. A similar pattern of results (temporal realignment after exposure to visual-leading asynchrony but not after exposure to auditory-leading asynchrony) was obtained using temporal order judgments (TOJs) instead of SJs (Experiment 3). Taken together, these results suggest that temporal recalibration still occurs for visual and auditory stimuli that fall clearly outside the so-called temporal window for multisensory integration and appear from different spatial positions. This temporal realignment may be modulated by long-term experience with the kind of asynchrony (vision-leading) that we most frequently encounter in the outside world (e.g., while perceiving distant events).

## Introduction

Experiencing asynchrony between visual and auditory signals that refer to the same event is more the rule than the exception (due to the fact that light travels 874,030 times faster than sound through the air). In order to maintain coherent representations of audiovisually asynchronous events, our brain slightly realigns visual and auditory signals in time [1]–[10]. According to previous studies, this temporal realignment may help us to perceive signals from different modalities as being simultaneous, thus facilitating multisensory integration. A brief exposure (e.g., for 3 min) to asynchronies falling inside or near the borders of the so-called “temporal window for multisensory integration” appears to be enough to originate this sensory recalibration. The limits of this temporal window (ranging from approximately 50–80 ms, when audition leads, to 150–250 ms, when vision leads) have been repeatedly shown for different types of stimuli [11]–[13].

The fact that more asynchrony is tolerated when the visual signal precedes the auditory signal may well reflect an effect of prior knowledge regarding audiovisual asynchronies in the outside world: Distant visual signals often arrive earlier than their correlated auditory signals. Visual precedence can also be observed in speech (e.g., the lips close around 150 ms before the sound/b/is produced; see [14], [15]) as well as in other dynamic stimuli [16]. Quite surprisingly, the temporal realignment of visual and auditory signals does not seem to reflect this perceptual asymmetry between visual-leading (VA) and auditory-leading (AV) asynchronies (see [2]). Here, we investigated whether temporal recalibration can be observed after exposure to a large (700 ms) audiovisual asynchrony. The use of such a large asynchrony allowed us to compare possible temporal adaptation effects in two different conditions: A VA condition where the visual and the auditory signals could plausibly have a common multisensory origin (i.e., a distant event), and an AV condition, where a multisensory origin could hardly be assumed. To our knowledge, temporal adaptation to large asynchronies (e.g., 400 ms) has only been tested once before; in a pilot study (including data from only 3 participants) reported by Fujisaki and colleagues [2]. A visual inspection of the data and graphic material of this study (motivated by the absence of reported statistics) may suggest that temporal recalibration would indeed be present after exposure to large asynchronies, especially in the case where the auditory signal preceded the visual signal during adaptation (see [2]). As this would contradict the idea of temporal adaptation to large asynchronies being more prevalent in “ecologically-valid” situations (with the visual signal leading), we further addressed this hypothesis.

Crossmodal associations can still be established out of the temporal window for integration by means of perceptual and associative learning. Experience makes us anticipate the sound of thunder some time after seeing its corresponding lightning. However, the link between visual and auditory stimuli can also be constructed not only for stimuli that appear at different points in time, but also for stimuli that appear from different spatial positions (e.g., hearing a specific sound followed by the appearance of food in the classical Pavlovian conditioning; see [17]). Although spatial coincidence has been observed to be crucial for multisensory integration in many studies using animal electrophysiology (see [18]) or spatial tasks (e.g., eliciting orienting responses) in human adults (see [19]), it appears to be less relevant for multisensory binding in tasks with no explicit spatial component (e.g., [20]). Keeping this evidence in mind, we studied whether temporal recalibration can be observed between largely-asynchronous signals (Experiment 1), but also between signals appearing from distant points in both time and space (Experiments 2 and 3).

Previous evidence suggests that several “temporal recalibrations” can co-occur for different spatial locations [21], [22]. This could perhaps indicate that temporal recalibration is specific for a particular spatial location (allowing for concurrent recalibrations taking place at different spatial positions). However, the spatial separation between visual and auditory stimuli does not seem to affect temporal recalibration in less “cluttered” experimental set ups where only 2 asynchronous stimuli are presented [20]. In the present study, we examined whether temporal recalibration occurs in a situation not yet studied in depth: When the visual and the acoustic stimuli appear very far apart in time and space, but paired in a consistent and continuous manner. Considering that an associative link can be established between relatively distant stimuli, evidence of temporal recalibration in both VA and AV conditions could be expected between spatiotemporally distant audiovisual signals. However, unidirectional realignment could be expected (only in the VA condition) assuming the hypothesis of temporal recalibration of large visual-leading asynchronies being governed by an experience-based prior suggesting a common distant multisensory origin.

Pairs of visual and auditory stimuli were presented asynchronously from the same spatial position (Experiment 1) or else from different positions (Experiments 2 and 3), for 3–4 min. A 706 ms silent gap, falling well outside the temporal window for multisensory integration (see [11], [12]) was introduced between the visual and the auditory stimuli, thus preventing synchrony perception (and, eventually, multisensory integration). Possible temporal recalibration effects were analyzed by comparing participants’ audiovisual simultaneity judgments (SJs; in Experiments 1 and 2) or temporal order judgments (TOJs; in Experiment 3) after exposure to audiovisual asynchrony (with either vision or audition leading) with SJs (or TOJs) taken after baseline exposure to audiovisual synchrony.

### Ethics Statement

Experiments 1, 2 and 3 were non-invasive, were conducted in accordance with the Declaration of Helsinki, and had ethical approval from the Parc Sanitari Sant Joan de Déu’s Ethics Committee. The participants provided a written informed consent to participate in the study.

## Experiment 1

### Materials and Methods

#### Participants

Sixteen experimentally-naïve participants (11 female, mean age of 21 years), with normal hearing, and normal or corrected-to-normal vision took part in the study and received 8 euros for their participation.

#### Procedure and materials

The experimental session consisted of an initial baseline/synchrony block, followed by an asynchrony block. Each block contained an initial exposure phase and a test phase. During the *exposure phase*, 80 pairs of audiovisual stimuli including a 250 Hz pure tone and a visual stimulus (a red round; diameter = 2.5 cm), both lasting 40 ms, were presented every 2536 ms. Participants sat approximately 50 cm in front of a computer monitor. The visual stimuli always appeared 1 cm above a continuously- and centrally-presented fixation point (a yellow LED) on a CRT monitor screen (Hyundai Q770, South Korea; 17′′; refresh rate = 75 Hz). The auditory stimuli were presented at 85db via 2 loudspeaker cones (Altec Lansing V52420, China), one placed on either side of the computer monitor at a distance of 21 cm with respect to fixation point. The visual and the auditory stimuli were presented by a PC in synchrony, in the first block, and asynchronously, in the second block. The participants were divided into 2 different groups (the *VA group* and the *AV group*) according to the ‘direction’ of the asynchrony perceived during the second exposure phase. In order to ensure that participants were attending to the visual stimuli during the exposure phase, a secondary oddball detection task was introduced during the exposure phase. The participants had to press a button every time they detected a target oddball stimulus (a smaller red round; diameter = 1.5 cm) that appeared only in 10% of the cases. Participants exhibited a near-perfect performance on this control task (1.95% of errors).

In the *test phase* (run immediately after the exposure phase), the participants performed SJs regarding auditory and visual stimuli that were identical as in the exposure phase and were randomly presented at different stimulus onset asynchronies (SOAs; ±320 ms, ±180 ms, ±80 ms, ±40 ms, or 0 ms; negative values = audition-first). Eight re-exposure audiovisual pairs of stimuli (identical as in the exposure phase) were presented following every 3 SJ trials, until 12 SJs were performed at each SOA. The experimental session lasted about 45 min and participants received 8 euros in exchange for taking part in the experiment.

In order to ensure that the asynchronous stimuli could not be perceived as being simultaneous after a 3–4 min of exposure, an independent group of 5 volunteers received a 3 min exposure to the same 706 ms (large) VA asynchrony as the one to be used in the experiment. In another block, the volunteers were exposed to a shorter asynchrony condition (220 ms). The order of exposure to the long and the short asynchronies was counterbalanced across participants. When asked, 2 of the volunteers perceived the audiovisual stimuli as being simultaneous after just 30 s of exposure to the short 220 ms asynchrony. All of the volunteers judged the audiovisual stimuli as being simultaneous after 3–4 min of exposure to the short asynchrony (showing indirect evidence of temporal recalibration). In contrast, the audiovisual stimuli in the large asynchrony condition (706 ms) were perceived asynchronously, by all of the volunteers, both after 30 s and after 3–4 min of exposure. This was taken as an indication of the fact that the level of asynchrony (706 ms) used during the main experiment was, as expected, too wide to generate the subjective impression of simultaneity during the experiment.

### Results and Discussion

A 3-parameter Gaussian function (parameterized with the mean, width and height) was fit to data obtained from participants’ audiovisual SJs (after passing a Shapiro-Wilk normality test). The mean correlation coefficient between observed and fitted data was .96. Using these fit, we derived the point of subjective simultaneity (PSS; defined as the mean of the Gaussian distribution) and the just noticeable difference (JND; defined as the standard deviation of the Gaussian fit). The PSS refers to the relative point in time at which the visual and the auditory stimuli were more likely to be perceived as simultaneous, and the JND has traditionally been taken as a measure of participants’ accuracy at judging simultaneity. The participants’ PSSs and JNDs were compared after exposure to synchrony and asynchrony, in order to search for possible temporal realignment effects.

**Figure 1:**
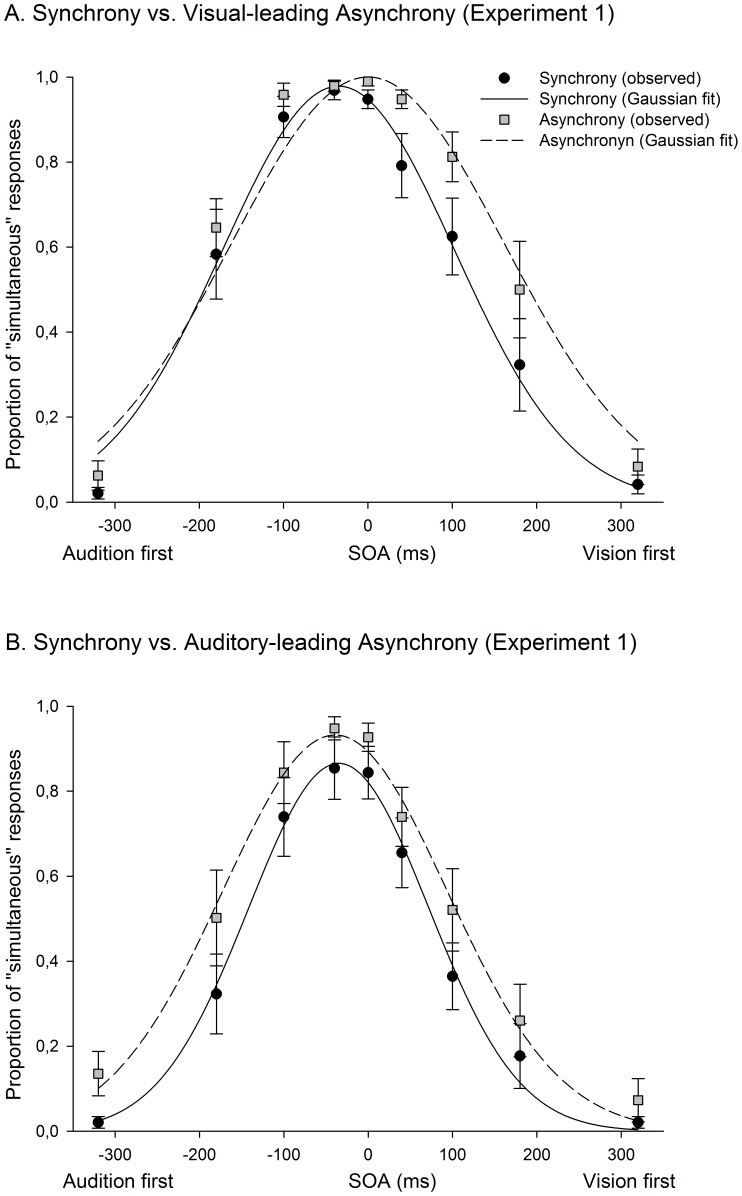
Results of Experiment 1 (spatial coincidence, SJ task). Proportion of participants’ ‘simultaneous’ responses plotted as a function of the stimulus onset asynchrony (SOA). The ‘simultaneous’ response probability for each SOA was computed for each participant, averaged across participants, and fitted into a Gaussian function for each condition. When the audiovisual stimuli were presented asynchronously during the exposure phase, a 23 **ms temporal shift was observed, in the point of subjective simultaneity (PSS), toward the direction of the asynchrony (vision-first). This effect was exclusively observed in the visual-leading condition (see Panel A), but not in the auditory-leading condition (Panel B). The SJ performance was significantly worse (i.e., larger JND) as a result of exposure to asynchrony in both conditions. Error bars indicate standard error**.

**Figure 2:**
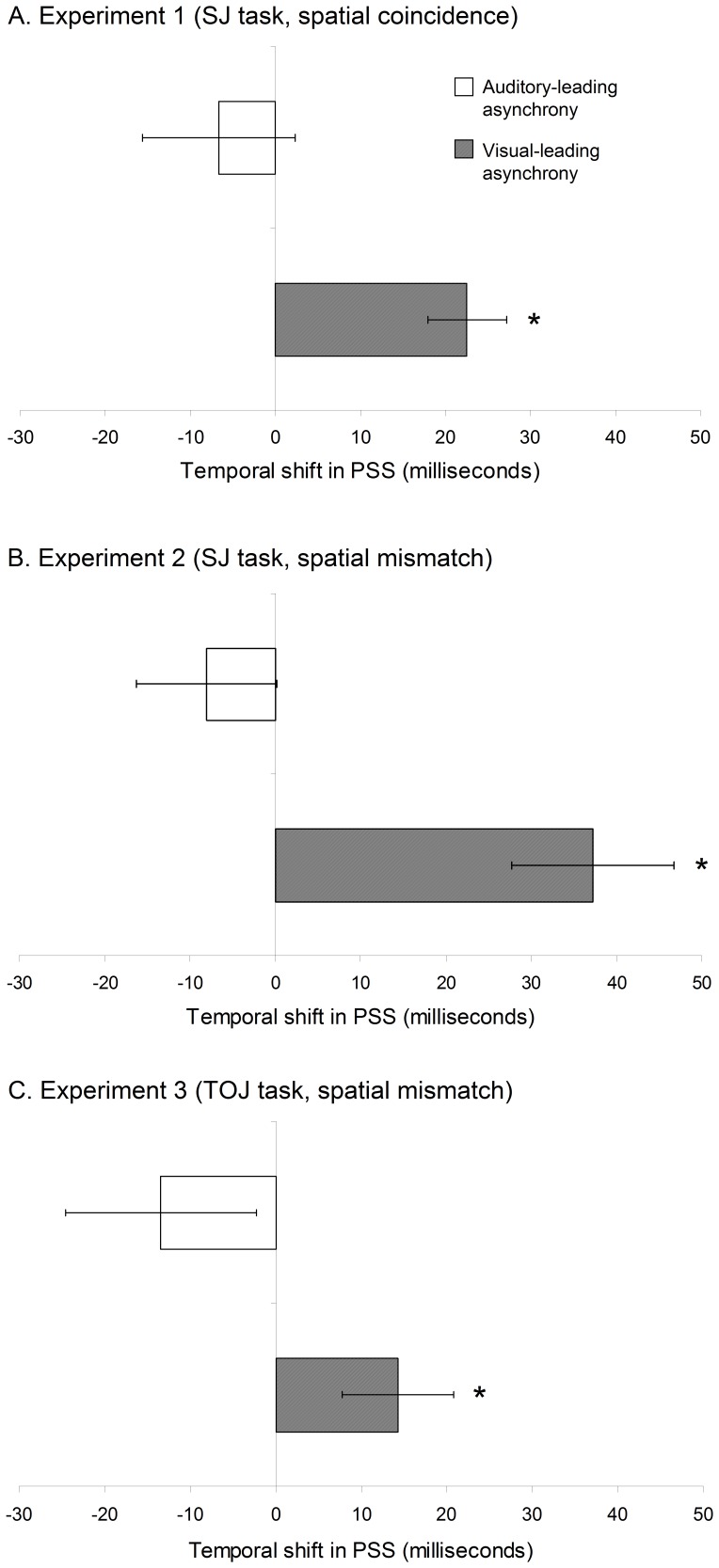
PSS effects across experiments. The bar graphs show the PSS group average for synchrony and asynchrony conditions in Experiments 1, 2, and 3. Significant effects shifts of the PSS were found after exposure to visual-leading (VA) but not auditory-leading (AV) asynchrony, in all of the experiments. Error bars indicate standard error.

A significant 23 ms shift of the PSS was observed (towards the exposure VA direction) after exposure to extreme audiovisual asynchrony, in the visual-leading (VA) group exclusively (see Figures 1 and 2A). An analysis of variance (ANOVA) conducted with PSS data, including the within-subjects factor ‘synchrony vs. asynchrony’ and the between-subjects factor ‘group’ (VA vs. AV), did not show a general difference between the conditions “synchrony” and “asynchrony” but revealed a significant interaction between the 2 factors (F(1,14) = 8.3, p = .01; Partial η^2^ = .372). Further analyses comparing synchrony and asynchrony revealed a significant temporal shift of the PSS in the direction of asynchrony in the group of participants that were exposed to VA asynchrony (*t*(7) =  −4.869, p = .002). In contrast, no difference between synchrony and asynchrony was observed in the AV group after the 3 min exposure to asynchrony (*t*(7) =  −737, p = .485). A subsequent ANOVA, including the same factors but JND data, revealed a significant increase of the JND after exposure to audiovisual asynchrony (F(1,14) = 6.906, p = .02; Partial η^2^ = .330); with no interaction between ‘synchrony vs. asynchrony’ and group (F(1,14) = .149, p = .705). Participants’ performance in the oddball detection task was very accurate (1.9% of errors).

These results suggest that temporal realignment between visual and auditory signals can be observed in conditions where subjects do not reach a subjective impression of audiovisual simultaneity. Although a decrease in accuracy in judging simultaneity was observed after exposure to both VA and AV asynchrony, the shift of the PSS only appeared when the visual stimuli preceded the auditory stimuli. This pattern of results may perhaps indicate that the brain is more prone to readjust the processing time of visual and auditory signals when a visual stimulus continuously appears earlier than an auditory stimulus (as when perceiving a distant event) than when the auditory stimulus precedes the visual stimulus.

## Experiment 2

**Figure 3:**
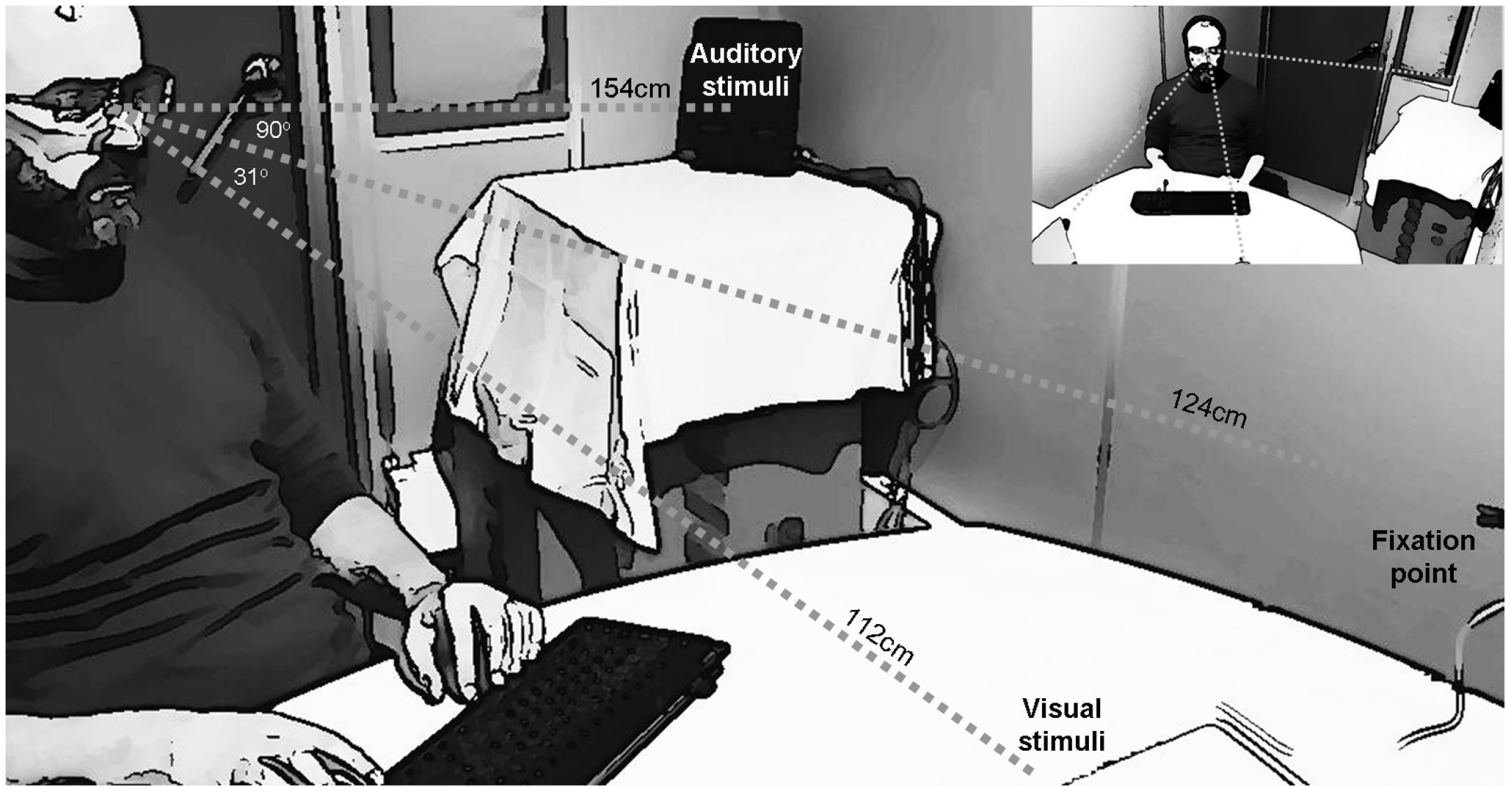
Experiments 2 and 3 set-up. The spatial positions of all stimuli presented during Experiments 2 and 3 (fixation point, visual and auditory stimuli) are shown from a side/large view and also from a frontal/small view. The participants were exposed, during an initial *exposure phase*, to synchronous (baseline) or else to asynchronous audiovisual stimuli. The visual and auditory stimuli were delivered from markedly different spatial locations. Unlike in the synchrony/baseline condition (where the participants received a 3–4 min exposure to visual and auditory stimuli presented simultaneously), a 706 ms silent gap repeatedly separated these 2 stimuli in the asynchrony condition. The possible after-effects of exposure to asynchrony were calculated, in a posterior *test phase*, by comparing simultaneity judgments (SJs) after the exposure synchrony (baseline) and asynchrony.

In order to investigate the possible influence of spatial separation on the temporal recalibration of large VA and AV asynchronies, we conducted an experiment where participants were exposed to visual and the auditory stimuli that appeared asynchronously from clearly different spatial positions (see Figure 3).

### Materials and Methods

#### Participants

Twenty-six experimentally-naïve participants (16 female, mean age of 23 years), with normal hearing, and normal or corrected-to-normal vision took part in the study and received 8 euros for their participation. Half of the participants were included in the VA group and the other half in the AV group.

#### Procedure and materials

The setup and procedure were the same as in Experiment 1 with the following exception: The visual and the auditory stimuli were presented from separate spatial positions during the whole experimental session (see Figure 3 for details).

### Results and Discussion

**Figure 4:**
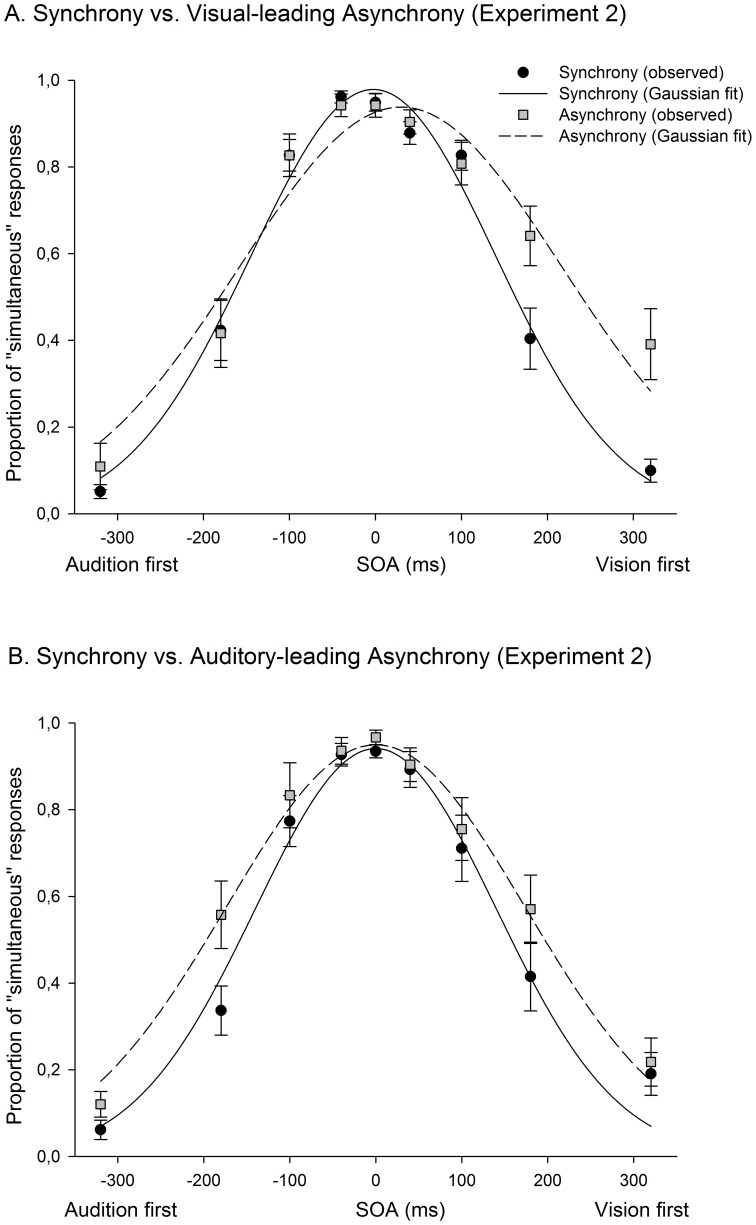
Results of Experiment 2 (spatial separation, SJ task). The PSS shifted 37 ms (in average) towards the direction of the asynchrony (vision-first) in the visual-leading condition (Panel A), but not in the auditory-leading condition (Panel B). Larger JNDs were observed after exposure to asynchrony in both conditions. Error bars indicate standard error.

Performance in the oddball detection task was very efficient (5% of errors). As in Experiment 1, the participants’ SJ responses in each SOA were fitted into a 3-parameter (height, width, and mean) Gaussian function. The mean correlation coefficient between observed and fitted data was .96. A 37 ms temporal shift was observed after exposure to extreme audiovisual spatiotemporal mismatch, in the VA group (see Figures 2B and 4). As in the previous experiment, no significant effect was observed in PSS after exposure to a large AV asynchrony. An ANOVA conducted with the PSS data (see Results of Experiment 1 for details) revealed a significant difference between synchrony and asynchrony (F(1,24) = 5.398, p = .029, Partial η^2^ = .184) and a significant interaction between the factors ‘synchrony vs. asynchrony’ and ‘group’ (VA vs. AV; F(1,24) = 12.947, p = .001; Partial η^2^ = .350). More detailed analyses showed a significant temporal shift of the PSS in the direction of asynchrony in the VA group (*t*(12) =  −3.937, *p* = .002) and no difference between the synchrony and asynchrony conditions in the AV group (*t*(12) = −.967, *p* = .353). A second ANOVA including JND data revealed that the JND increased after exposure to audiovisual asynchrony (F(1,24) = 9.774, p = .005; Partial η^2^ = .289); with no interaction between ‘synchrony vs. asynchrony’ and group (F(1,24) = 1.1, p = .305).

A subsequent ANOVA was carried out, separately for VA and AV groups, including PSS data and the conditions ‘synchrony vs. asynchrony’ and ‘experiment’ (Experiment 1 vs. Experiment 2), to compare the results obtained in Experiments 1 and 2. These analysis revealed a significant difference between the synchrony and asynchrony conditions in the VA group (F(1,19) = 22.207, p = .00015; Partial η^2^ = .539) and no interaction between the 2 factors (F(1,19) = 1.332, p = .263; Partial η^2^ = .066), suggesting that the shift observed in the VA groups was equally large when the visual and auditory stimuli were presented from the same (Experiment 1) or from different locations (Experiment 2) during exposure and test phases. Further analyses conducted with PSS data from Experiments 1 and 2 confirmed the lack of temporal shift in the AV groups of both experiments (F(1,19) = 1.362, p = .264; Partial η^2^ = .065), and the absence of interaction between the factors ‘synchrony vs. asynchrony’ and ‘experiment’ (F(1,19) = .11, p = .916; Partial η^2^ = .001).

This pattern of results reinforces the idea that temporal recalibration not only arises as a result of exposure to asynchronies in (or nearby) the temporal window for audiovisual integration, but also as a consequence of perceiving large asynchronies. The results of the Experiment 2 also confirm that this adaptation only occurs for visual-leading asynchronies. As in Experiment 1, experiencing audiovisual asynchrony increased the JND in both VA and AV conditions. In line with another previous study by Keetels and collaborators [20], our results suggest that the spatial separation has virtually no effect on temporal recalibration in cases where spatial co-occurrence is not crucial for integrating/grouping the visual and the auditory signals (see [10]). It is worth highlighting that the amount of temporal and spatial separation was deliberately laid down to avoid multisensory apparent motion (e.g., see [23]). The overlap between the results of Experiments 1 (in which visual and auditory stimuli appeared from approximately the same position in space) and 2 also indicate that the results of Experiment 2 were not the consequence of an uncontrolled influence of apparent motion.

However, PSS shifts can, according to some authors (e.g., see [24]), not only reflect genuine temporal shifts between sensory modalities, but also a bias in the response criterion. The fact that the PSS shifts, in Experiments 1 and 2, is observed in one of the conditions (VA) but not in the other (AV) could be easily taken as an argument against this possible interpretation of our results: Why should response bias affect participants’ SJs in one condition but not in the other? Experiment 3 was designed to clarify this issue further.

## Experiment 3

In order to see whether the results of Experiments 1 and 2 could be replicated using another task or not, we conducted a third experiment in which participants performed temporal order judgments (TOJs) instead of SJs. Previous studies evidenced the fact that the processes (and perceivers’ possible biases) are partially different in these two tasks (e.g., see [24]–[26]). Therefore, replicating the evidence found in Experiment 2 with TOJ would strongly support the idea that temporal recalibration arises between stimuli that are very distant with respect to each other in both space and time.

### Materials and Methods

#### Participants

Forty-two experimentally-naïve participants (26 female, mean age of 24 years), with normal hearing, and normal or corrected-to-normal vision took part in the study and received 8 euros for their participation. As in the previous experiments, the participants were divided into two different groups; VA and AV.

#### Procedure and materials

The setup and procedure remained the same as in Experiment 2 (see Figure 3) with the following exception: A TOJ task was used instead of an SJ task.

### Results and Discussion

**Figure 5:**
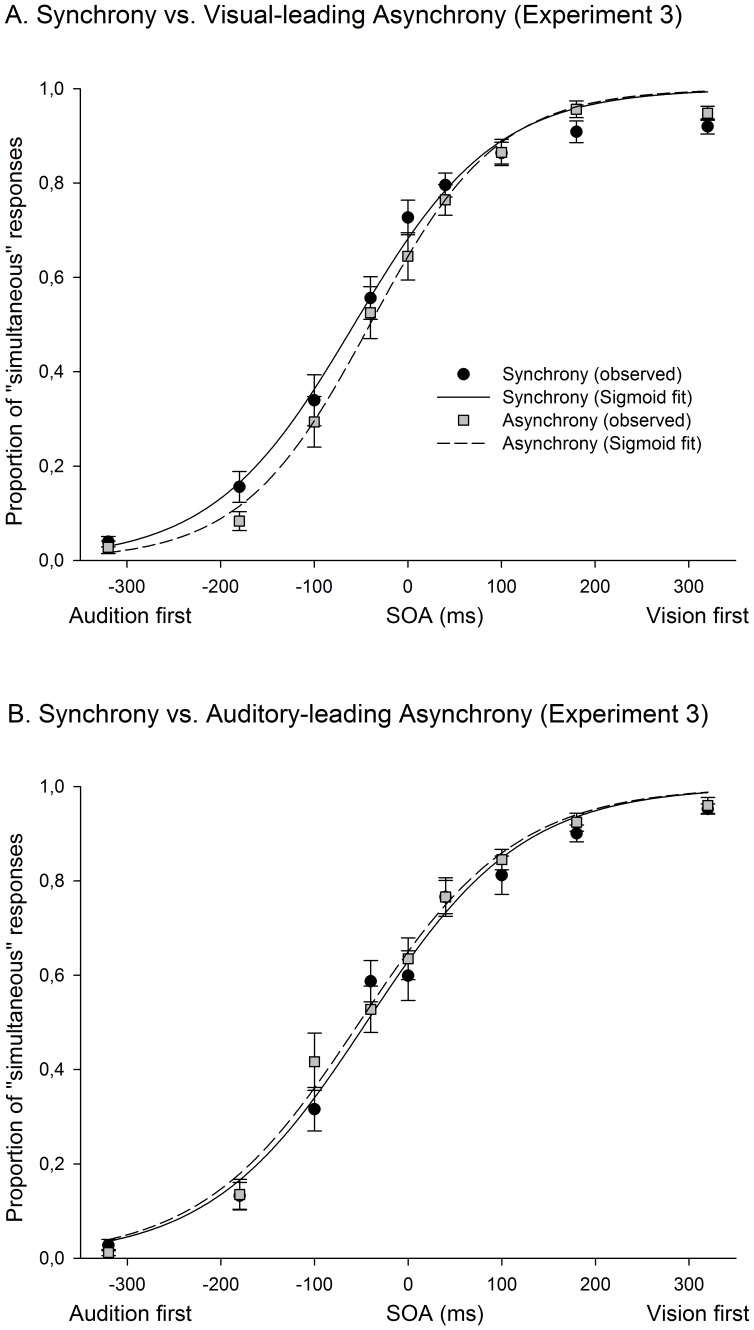
Results of Experiment 3 (spatial separation, TOJ task). As in the previous experiments, a significant 14 ms temporal shift was observed towards the direction of the asynchrony only in the visual-leading (VA) group (see Panel A). The difference in PSS between the synchrony and the asynchrony conditions was not significant in the AV group (Panel B). Error bars indicate standard error.

Participants were very accurate at detecting oddball visual stimuli (control task) during the exposure phase (1.28% of errors). Data obtained from participants’ audiovisual TOJs were fitted into a 3-parameter (including height, width, and mean) Sigmoid function (mean correlation coefficient between observed and fitted data = .96) in order to derive PSS (i.e., the mean of the Sigmoid distribution) and the JND (i.e., the width of the distribution). Note that the scale differences across experiments of our indicators of JND were irrelevant for the comparisons between the synchrony and asynchrony conditions. As observed in the previous two experiments, only the VA group exhibited a shift of the PSS after exposure to audiovisual spatial and temporal mismatch (see Figures 2C and 5). An ANOVA failed to show a significant difference in PSS between synchrony and asynchrony (F(1,40) = .004, p = .947, Partial η2 = .001), but revealed a significant interaction between the factors ‘synchrony vs. asynchrony’ and ‘group’ (F(1,40) = 4.607, p = .038; Partial η2 = .103). Paired comparisons ran in the VA and the AV group separately revealed a significant PSS shift in the direction of asynchrony (see figures 4 and 5) in the VA group (t(20) =  −2.191, p = .04) and no difference between the synchrony and asynchrony conditions in the AV group (t(20) = 1. 205, p = .242). A second ANOVA failed to show a difference in JND between the synchrony and the asynchrony conditions (F(1, 40) = 1.4, p = .244) or an interaction between the 2 groups (VA and AV; F(1, 40) = .365, p = .546).

A third ANOVA, conducted with data from the VA groups and comparing the results obtained in Experiments 2 and 3 (thus including the factors “synchrony vs. asynchrony” and “experiment”), evidenced a significant general difference between the synchrony and asynchrony conditions in this group (F(1,32) = 21.425, p = .000058; Partial η2 = .401) and a significant interaction between the 2 factors (F(1,32) = 4.4239, p = .048; Partial η2 = .117), suggesting that the shift observed in Experiment 2 (37 ms) was larger than the shift observed in Experiment 3 (14 ms). It is worth highlighting that, unlike in the AV group, the PSS shift was consistently observed, towards the exposed direction (vision-first), in the majority of the participants in the VA group (16 out of 21) in Experiment 3.

The results of Experiment 3 confirmed, by means of a TOJ task instead of an SJ task, that a 3-min exposure to a large audiovisual asynchrony induces temporal recalibration between the exposed visual and auditory signals. This effect was present, in both Experiment 2 and 3, for stimuli presented at different spatial positions. However, the amount of temporal shift seemed to be, in line with previous literature [25], larger in participants that performed SJs (Experiment 1) than in participants that performed TOJs (Experiment 3; see Figure 2).

## General Discussion

In three different experiments, we addressed the two main questions of the present study: (1) Does temporal realignment between visual and auditory signals still occur when the conditions for multisensory integration (i.e., approximate coincidence in space and time) are not met? And (2) is there an asymmetry between VA and to AV large asynchronies in terms of temporal recalibration? The latter question was motivated by the fact that large asynchronies (e.g., when perceiving events from afar) tend to be perceived in one direction (VA), but not in the other (AV), as a consequence of the difference in velocity of transmission in the air between light and sound.

A general interpretation of our results may be that temporal recalibration is generated whenever a constant temporal link between visual and auditory stimuli is established (in this order of appearance) even beyond the limits of the temporal window for audiovisual integration. Temporal recalibration was observed, in the present study, only after experiencing VA asynchrony. Although the absence of an effect in the AV condition may contradict previous studies reporting temporal realignment after a short-term exposure to asynchrony (see [1]–[9]), this contradiction is only apparent. An obvious difference between the present and the majority of previous studies resides in the size of the asynchrony used: While relatively short asynchrony intervals – falling either inside or near the temporal window for integration – were used in most of the previous studies, a large (706 ms) audiovisual asynchrony was used here to impede the subjective construction of a synchronous multisensory percept.

Our results may also be in apparent contradiction with a previous pilot study (including data from a much reduced sample) published by Fujisaki and colleagues [2]. While larger temporal shifts can be observed (by visual inspection motivated by the absence of reported statistics) in the AV condition than in the VA condition in this previous study, the opposite pattern was found here in 3 different experiments. Keeping in mind that VA large asynchronies are commonly experienced on a daily basis (as they are generated by the distance between the perceiver and the perceived event), and also that that large auditory-leading asynchronies are hardly found in the environment, our findings (but perhaps not the preliminary results in [2]) may well be interpreted as a consequence of prior experience. Previous research has shown that our prior perceptual experience can strongly influence the temporal aspects of audiovisual integration (e.g., [27]). Prior experience with audiovisual asynchronies would, therefore, make it easier for the brain to assume a multisensory common origin of the visual and the auditory signals when experiencing a VA large asynchrony. The fact that more asynchrony is tolerated in this particular asynchrony direction (VA) than in the other (see [11], [12]) would perhaps reinforce the idea that the brain adapts (arguably through experience) to the perceptual constraints imposed by the environment. The visual-leading specificity of the adaptation to large asynchronies could easily be seen as another example of how prior knowledge influences basic aspects of human perception.

In another previous study conducted by Vroomen and colleagues [9], a 350 ms asynchrony interval was used in a ‘control’ experiment to see whether temporal recalibration arises between audiovisual stimuli presented immediately outside the temporal window for audiovisual integration (see footnote 1). Although no significant differences were reported between this experiment and another experiment showing temporal realignment after exposure to shorter asynchrony lags (e.g., 200 ms), the authors reported that exposing to 350 ms asynchrony had no effects on PSS and concluded that temporal recalibration is not possible beyond certain temporal limits (arguably beyond the temporal window for audiovisual integration). The pattern of results presented here may perhaps disprove this idea for the case of exaggerated visual-leading asynchronies. It is worth highlighting that previous evidence may also contradict Vroomen et al.’s conclusion even for asynchronies bordering the window for integration (see Figure 2B in [2]).

Similarly as in [20], we observed a shift in the perceived simultaneity even for visual and auditory signals that appeared from different spatial positions. Considering recent evidence of temporal adaptation to different audiovisual asynchronies co-occurring at different spatial positions [21], [22] together with Keetels et al.’s [20] and our results, a possible conclusion may be that the spatial specificity of this adaptation can be accommodated to different scenarios: While multiple (and spatial-specific) temporal recalibrations can be found when perceiving 2 nearly-concomitant audiovisual asynchronies, space does not seem to matter for resynchronization when single visual and auditory stimuli are presented asynchronously from different spatial positions. Further research is needed to elucidate whether the processes involved in temporal recalibration are the same or not, and involve the same areas of the brain or not, depending on each perceptual situation (i.e., when perceiving multisensory signals that appear asynchronously from the same or different spatial positions; or when visual and auditory signals fall inside, nearby or outside the temporal window for audiovisual integration).

A temporal realignment of the PSS was observed, in the present study, using both SJ and TOJ. However, this effect was significantly larger in participants judging simultaneity than in participants deciding the order of appearance of the visual and the auditory stimuli. This result fits well with previous evidence suggesting less stable [2] or even null [25] temporal adaptation effects as delivered from TOJs. Although investigating the differences between SJ and TOJ was not the main focus of the present work, we would like to emphasize that these 2 tasks depend, although not completely, on different perceptual/attentional and decisional processes (see [24]–[26] for details). Importantly, the fact that the same pattern of results was obtained using either SJ (Experiments 1 and 2) or TOJ (Experiment 3) and different experimental setups (with spatial separation between the adapted/tested stimuli, in Experiments 2 and 3, or not, in Experiment 1) strongly supports the idea that participants’ responses were influenced by a genuine temporal realignment, and not only by other higher-level and task-depending factors. Furthermore, no temporal realignment was observed, as one would expect following a pure “response bias” account of the results, in the AV condition (in 3 different experiments).

The JND results were less consistent than the PSS results across the 3 experiments. An increase of the JND observed after exposure to both VA and AV asynchronies was observed in Experiments 1 and 2 (where a SJ task was used). In contrast, this effect was not observed in Experiment 3 (TOJ task). As several previous studies already pointed out (e.g., [24]) an increase of the JND can have different causes. One plausible explanation of the effects found in Experiments 1 and 2 may be that the repeated exposure to contingent visual and auditory stimuli increases the probability for them to appear together. This could make it harder to ‘separate’ the visual and the auditory signals in time, as consequence of an intensification of the intersensory ‘unity assumption’ (see [28], [29]). Another possibility may be that the adaptation phase relaxed the criterion for responding ‘synchronous’. Unfortunately, the experimental paradigm used in the present study does not allow us to clarify whether the adaptation to asynchrony broadly decreased the participants’ temporal sensitivity or else relaxed their criterion for giving a “synchronous” response. The temporal sensitivity effects may well depend, in the present and in previous studies, on multiple factors, being subject of speculation. Further research will be needed to determine the exact origin of the JND increment observed here and in several previous studies (see [2], [30]).

### Conclusions

The present findings suggest that audiovisual temporal recalibration can take place beyond the temporal limits of multisensory integration and for sensory signals appearing from different spatial positions. The everyday experience with spatiotemporally-separated visual and auditory signals seems to have a huge impact on the way these signals are perceived: Exposure to visual-leading (but not auditory-leading) asynchrony induces temporal realignment. Taken together, our results reflect the significant role that short- and long-term experience has in shaping basic perceptual mechanisms.
